# Molecular Identification and Traceability of Illegal Trading in* Lignobrycon myersi* (Teleostei: Characiformes), a Threatened Brazilian Fish Species, Using DNA Barcode

**DOI:** 10.1155/2016/9382613

**Published:** 2016-09-07

**Authors:** Alexandre dos Santos Rodrigues, José Henrique Souza Galdino Brandão, Jamille de Araújo Bitencourt, Ricardo Jucá-Chagas, Iracilda Sampaio, Horácio Schneider, Paulo Roberto Antunes de Mello Affonso

**Affiliations:** ^1^Department of Biological Sciences, Universidade Estadual do Sudoeste da Bahia, Rua José Moreira Sobrinho, s/n, Bairro Jequiezinho, 45206-190 Jequié, BA, Brazil; ^2^Institute of Coastal Studies (IECOS), Universidade Federal do Pará, Alameda Leandro Ribeiro s/n, Bairro Aldeia, 68600-000 Bragança, PA, Brazil

## Abstract

*Lignobrycon myersi* is a threatened freshwater fish species and endemic of a few coastal rivers in northeastern Brazil. Even though the Brazilian laws prohibit the fisheries of threatened species,* L. myersi* is occasionally found in street markets, being highly appreciated by local population. In order to provide a reliable DNA barcode dataset for* L. myersi*, we compared mitochondrial sequences of cytochrome c oxidase subunit I (COI) from fresh, frozen, and salt-preserved specimens. Phylogenetically related species (*Triportheus* spp.) and other fish species (*Astyanax fasciatus*) commonly mixed with* L. myersi* in street markets were also included to test the efficiency of molecular identification. In spite of the differences in conservation processes and advanced deterioration of some commercial samples, high-quality COI sequences were obtained and effective in discriminating* L. myersi* specimens. In addition, while populations from Contas and Almada River basins seem to comprise a single evolutionary lineage, the specimens from Cachoeira River were genetically differentiated, indicating population structuring. Therefore, DNA barcoding has proved to be useful to trace the illegal trading of* L. myersi* and to manage threatened populations, which should focus on conservation of distinct genetic stocks and mitigation on human impacts along their range.

## 1. Introduction

The hydrographic coastal basins in Eastern Brazil are characterized by high endemism of aquatic species [1] with an alarming number of critical areas (about 46% of the total region) for conservation of fish species [2]. These areas combine endemic species, advanced deforestation, overexploitation, and introduction of invasive species, being particularly predominant in drainage of northeastern Brazil [2, 3]. The species* Lignobrycon myersi* Miranda Ribeiro, 1956, perfectly exemplifies this situation. This small fish species (up to 11 cm in total length) is endemic of southern Bahia in northeastern Brazil, formerly described only from a single stream from Almada River basin [4]. About two decades later, this species was found in Contas River basin close to Jequié, one of the largest cities in southern Bahia [3], and a few specimens have been recently collected in Cachoeira River basin [1]. Because of their restricted range (about 200 km) and habitat modifications (Contas and Cachoeira Rivers are highly polluted while invasive carnivore fishes have been introduced in the three basins),* L. myersi* was included in Red List of Threatened Species of Brazilian Fauna [3, 5].

The fisheries and trading of threatened species are illegal according the Brazilian laws [6], but monitoring of fish products is largely ineffective in street markets in several regions of Brazil [7]. Therefore, specimens of* L. myersi* have been traditionally collected and commercialized with other morphologically similar and small characins (e.g., species of* Triportheus* and* Astyanax*) in street markets of southern Bahia, northeastern Brazil (pers. obs.). After being eviscerated, the whole specimens are fried and served as a local and highly appreciated appetizer, called “pititinga.” This practice hinders the precise identification of fish products based on external morphology thus favoring the commercialization of threatened species in markets across the region.

In this sense, forensic genetics represents a reliable approach to minimize the frauds in fish products from endangered species related to nondeclared or mislabeled fisheries [8]. Besides being useful for identifying wildlife, DNA analysis is also able to discriminate local fish stocks and overexploitation of specific populations [9–11]. Over the last years, subunit I of the cytochrome c oxidase (COI) has been successfully applied to establish interspecific molecular divergence inasmuch as they provide a species-specific DNA barcode [12–15].

In the present paper, COI sequences were analyzed to identify specimens of* Lignobrycon myersi* throughout their range and commercialized in street markets of southern Bahia, representing the largest DNA sample obtained for this species so far. In order to evaluate whether the quality of muscular tissue could interfere in the reliability of molecular data or not, frozen and salt-preserved market samples were compared to the tissue of fresh and recently collected specimens. In addition, the same DNA regions were sequenced from other phylogenetically related species and other characins commonly commercialized with* L. myersi* to test the utility of COI as DNA barcodes.

## 2. Materials and Methods

### 2.1. Sampling

**Figure 1:**
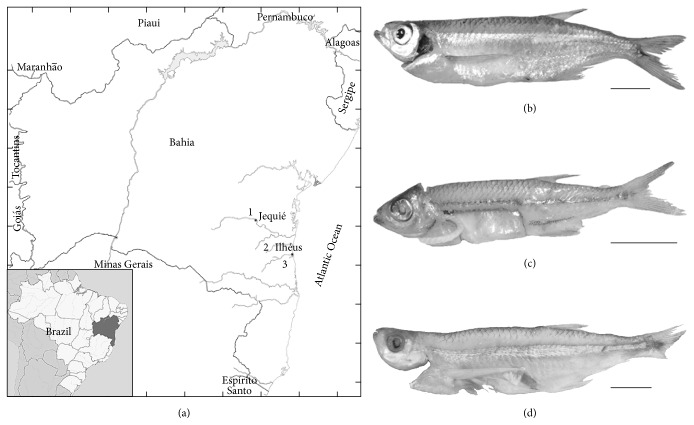
Map of the state of Bahia in northeastern Brazil (a), indicating the three collection sites (1: Contas River, 2: Almada River, and 3: Cachoeira River) of* Lignobrycon myersi*. On the right, photographs of fresh (b), frozen (c), and salt-conserved (d) specimens of* L. myersi* (the bar equals 1 cm).

A total of 41 samples of muscle tissue (nearly 0.5 cm^3^) of* L. myersi* from Pedra reservoir in Contas River, Jequié, Bahia, were obtained from local fishermen at street markets. These samples have been frozen for undetermined periods or else covered with salt and exposed to room temperature (about 20 to 35°C) along with other small fish species (Figure 1). For comparative analyses, tissue samples of recently collected specimens of* L. myersi* from the type-locality in Almada River basin (*n* = 14) and Cachoeira River basin (*n* = 4), Ilhéus, Bahia, were included in the molecular analyses, thus representing the total range of this species (Figure 1). Voucher specimens were deposited in the fish collection of Instituto Nacional da Mata Atlântica (INMA) in Santa Teresa, State of Espírito Santo (#MBML6400). Because of their close phylogenetic relationship with* Lignobrycon* [16, 17], COI sequences of* Triportheus* species obtained in the present study and available in GenBank were included. These additional sequences comprised* Triportheus guentheri* from São Francisco River (present paper; HM404957.1) [18],* T. nematurus* (GU701461.1; GU701945.1; GU701458.1; GU701457.1) [19], and* T. angulatus* (GU060427.1) [20]. Moreover, specimens of* Astyanax fasciatus* from Contas River basin, usually found mixed with* L. myersi* individuals in markets under the denomination of “piabas,” were also analyzed.

### 2.2. Amplification and Sequencing of COI Fragments

Total DNA was extracted from muscle tissue using the kit Wizard® Genomic DNA Purification (Promega) according to manufacturer's instructions. The amount of integrity of extracted DNA was checked after electrophoresis in 0.8% agarose gel stained with GelRed*™* and visualization under UV light. Even though the commercial samples presented highly degraded DNAs, we estimated a mean DNA concentration of 50 ng/*μ*L. The COI sequences were amplified via PCR using the primers: VF1i_t1 5′-TGTAAAACGACGGCCAGTTCTCAACCAACCACAAAGACATTGG-3′ and VR1i_t1 5′-CAGGAAACAGCTATGACTAGACTTCTGGGTGGCCAAAGAATCA-3′ [21].

The PCR conditions comprised a final concentration of 0.2 mM dNTP, 1x buffer, 2 mM MgCl_2_, 0.4 *µ*M of each primer, 1 U of* Taq* DNA polymerase, 50 ng of template DNA, and ultrapure water to a final volume of 15 *μ*L. The amplification program encompassed a first denaturation step at 94°C for 5 min, followed by 30 cycles at 94°C for 40 sec, 55°C for 40 sec, 72°C for 30 sec, and final extension at 72°C for 7 min.

The sequencing reaction was carried out with the kit Big Dye (ABI Prism*™* Dye Terminator Cycle Sequencing Reading Reaction, Applied Biosystems) to a final volume of 10 *µ*L, using 2.0 *µ*L of each primer (0.8 pmol/*µ*L), 1 *µ*L of PCR product, 1 *µ*L of Big Dye mix, 1.5 *µ*L of buffer, and 4.75 *µ*L of ultrapure water. Sequences were obtained in a Prism 3500XL ABI (Applied Biosystems) automatic sequencer.

### 2.3. Sequence Analyses

The sequences were edited in the software BioEdit Sequence Alignment Editor 7.1.9 [22] and aligned using ClustalW [23] also available in BioEdit. Afterwards, they were submitted to BLAST [24] to confirm and compare the sequenced regions with similar sequences available in GenBank (http://www.ncbi.nlm.nih.gov/).

The levels of intra- and interspecific diversity as well as the construction of trees using Neighbor-Joining (NJ) [25] according to Kimura-2-parameter (K2P) evolutionary model [26] were performed in the software MEGA v. 6 [27], as proposed in DNA barcoding. The branch support was established by bootstrap after 10,000 replications [28]. In addition, a COI tree based on Bayesian inference (BI) was built in the software MrBayes v. 3.1 [29], using the evolutionary model HKY+I, as determined by jModelTest [30]. In this case, the support of branches was estimated according to a posteriori probabilities.

The species delimitation was also estimated by barcode gap analysis using ABGD (Automatic Barcode Gap Discovery for primary species delimitation) in the platform http://wwwabi.snv.jussieu.fr/public/abgd/ and the Bayesian implementation of the PTP (bPTP) available in http://species.hits.org/ptp/. The bPTP model [31] adds Bayesian support values to the species identified from previous Maximum Likelihood obtained in MEGA v. 6 [27]. In this study, the bPTP settings were rooted trees, 100,000 Markov Chain Monte Carlo (MCMC) generations (thin = 100), and burn-in of 10%.

Furthermore, a haplotype network based only on COI sequences of* L. myersi* was obtained using the software Haplotype Viewer [32] in order to evaluate putative differences among populations. The values of haplotype (*h*) and nucleotide (*π*) diversity were calculated with DNA Sequence Polymorphism, DnaSP v. 5.10 [33].

## 3. Results and Discussion

**Figure 2:**
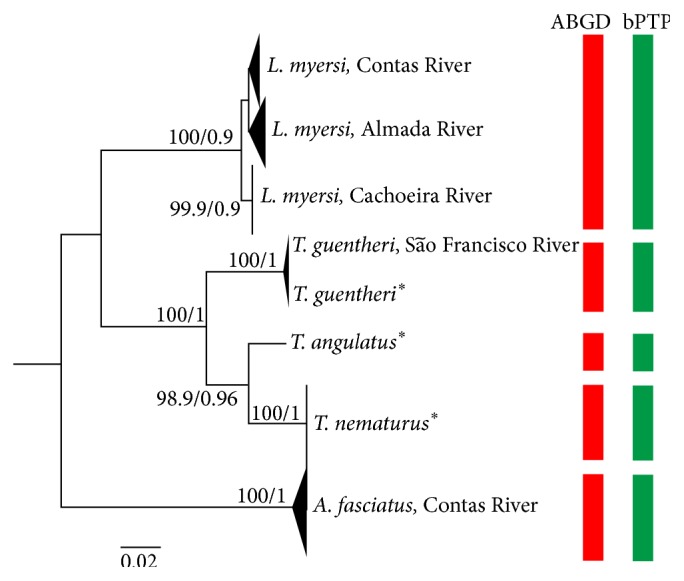
Consensus tree based on NJ and BI analysis of COI sequences in* L. myersi *from Contas (*n* = 41), Almada (*n* = 14), and Cachoeira (*n* = 4) River basins,* Triportheus guentheri* (*n* = 4),* T. angulatus* (*n* = 1),* T. nematurus* (*n* = 4), and* Astyanax fasciatus* (*n* = 3). *∗* indicates the sequences obtained in GenBank. The columns on the right indicate the five taxonomic entities (species) recovered by ABGD and bPTP algorithms.

The COI dataset for* L. myersi* comprised 59 sequences (662 bp), besides those obtained from* Astyanax fasciatus* (Contas River basin) and three species of* Triportheus*, totaling 71 COI sequences. The genetic divergence among the three genera ranged from 17.8% to 24.1%, with a mean value of 21.3% (Table 1). As a result, the consensus tree based on NJ and BI methods (Figure 2) formed three main clusters that discriminate* Lignobrycon*,* Triportheus* spp., and* Astyanax fasciatus* with high support values (100% of bootstrap and 0.9–1 of a posteriori probability). The highest differentiation between* Astyanax* and the other genera (22 to 24%) was expected because* Lignobrycon* and* Triportheus* have been recently removed from the family Characidae and placed into a distinct family (Triportheidae) according to phylogenetic analyses [16]. Indeed,* L. myersi* shares several synapomorphic traits with* Triportheus*, including an unusual sex chromosome system in Characiformes [17].

Moreover, five reciprocal monophyletic clusters were reliably recovered in the consensus tree (Figure 2), corresponding to the nominal species. Similarly, both species delimitation algorithms (ABGD and bPTP) indicated the presence of five species in the dataset (Figure 2). Indeed, the values of interspecific distance ranged from 4.9 to 24.6% (Table 2) while the intraspecific divergence varied from 0 (*T. nematurus*) to 0.5% (*A. fasciatus*), with mean values of 0.2% (Table 3).

According to some authors [34], the minimum divergence values in COI sequences among the species should be, at least, 10 times higher than intraspecific variation to proper species identification. In this study, the genetic differences among species were, on average, 24.5 times higher than within species, characterizing the barcode gap. This result corroborates the efficiency of COI sequences in discriminating fish species [15, 19] with applications for taxonomy and forensic analyses as a reliable method of DNA authentication [35, 36] inasmuch as* L. myersi* was clearly distinguished from closely related species (*Triportheus* spp.) and from other species (*A. fasciatus*) commonly commercialized in regional markets along with* L. myersi* and other small characins under the same denomination (“piabas”).

It should be pointed out that even though degraded DNA samples were obtained from most of frozen and salt-preserved specimens, the sequencing results were invariably congruent in* L. myersi*, yielding high-quality COI sequences from small tissue samples. Therefore, DNA barcoding has proved to be advantageous to the traceability of commercial fish products [35]. Taking into account that molecular identification of species using COI is fast and reliable, this genetic marker can be used to identify specimens of* L. myersi* in markets or food products. Indeed, COI sequences were informative to species identification even when closely related species were analyzed, like* Triportheus* (Figure 2).

**Figure 3:**
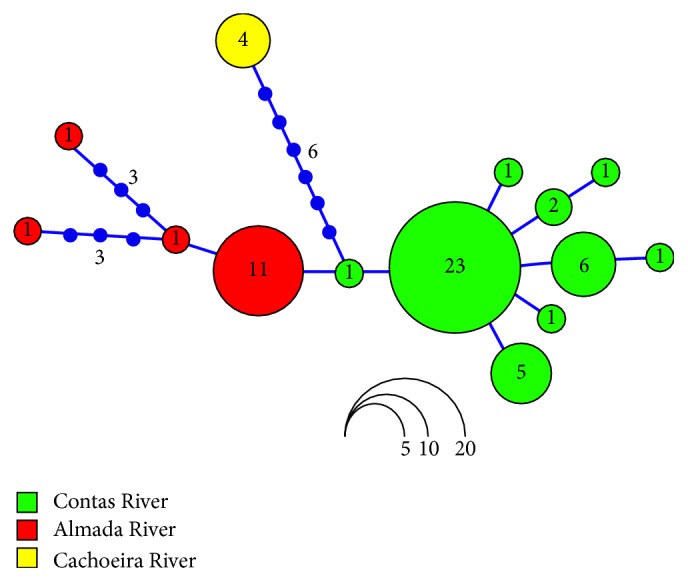
Haplotype network based on COI sequences from the populations of* L. myersi* (Contas, Almada, and Cachoeira River basins). Each color represents a river basin and the circle size is equivalent to the number of individuals (shown inside the circles) sharing the same haplotype. The values on each line indicate the number of mutations.

In addition, the COI analysis allowed differentiating the population of* L. myersi* from Cachoeira River (bootstrap = 99.9%; a posteriori probability = 0.9) (Figure 2), since this group presented a genetic distance of 1.3% in relation to samples from both Contas and Almada River (Table 4). Likewise, the haplotype network showed that the specimens from Cachoeira River are more genetically divergent, being separated from the other populations by six mutations (Figure 3). In fact, several DNA barcode studies in Neotropical ichthyofauna have detected high genetic divergence (>2%) within a single putative taxonomic entity [19, 37, 38]. In these cases, the authors are unanimous in stating that cryptic species not formerly detected by traditional taxonomy should be present.

Indeed, the genetic divergence of* L. myersi* from Cachoeira River, although below the 2% threshold used in fish barcoding and without differentiation by ABGD and bPTP algorithms (Table 4; Figure 2), suggests that this population is structured. From a conservation viewpoint, this result implies that the specimens from Cachoeira River should be managed as a unique and endangered stock, particularly because of the intensified deforestation of marginal areas and pollution of this basin [39]. From a biogeographic perspective, this result contradicts the putative similarity between Almada and Cachoeira River basins based on fauna composition, geographic distance, and chromosomal data [40, 41]. Otherwise, the evolution of coastal hydrographic basins in Bahia seems to be more complex, probably alternating periods of connectivity and isolation such as headwater capture, as demonstrated in other reports [41, 42].

Moreover, the levels of haplotype diversity (*h*) were also differentiated among populations, ranging from 0 (a single haplotype) in Cachoeira River to 0.60 in Contas River samples (Table 5). Nonetheless, this variation might be biased by sample size, once the population of Cachoeira River was represented by four specimens while 41 individuals were obtained from Contas River. On the other hand, the population from Almada River, though composed of 14 analyzed specimens, presented the highest nucleotide divergence (*π* = 0.002) (Table 5). In fact, this population is apparently larger and located on less impacted areas when compared to those from Cachoeira and Contas Rivers based on several field trips along the range of* L. myersi* over the last four years.

According to this observation, the population from Almada River seems to represent a suitable stock to genetic management of* L. myersi* with views to restoration or translocation of individuals, particularly in relation to Contas River, which probably corresponds to the most depleted population of* L. myersi* considering the illegal fishing and other human impacts such as introduction of exotic carnivore species, water contamination, and construction of dams [3].

## 4. Conclusions

We conclude that COI sequences are highly informative to the forensic genetics in the illegal trading of* L. myersi*, ensuring the detection of frauds or mislabeling of fisheries products even when morphological identification is impracticable.

This molecular approach is important for monitoring more precisely the impacts of overexploitation and genetic management of threatened fish species in northeastern Brazil and for supporting the application of law penalties against illegal fisheries. Nonetheless, this strategy should be accompanied by environmental education policies to awake the population about the regional biodiversity and reduce the demanding for the commercialization of* L. myersi*.

## Figures and Tables

**Table 1:** Genetic distance of COI sequences among genera based on K2P model.

Genus	Sample size (*n*)	1	2
(1) *Lignobrycon*	59	—	
(2) *Triportheus*	9	0.178	—
(3) *Astyanax*	3	0.220	0.241

*Mean*		*0.213*	

**Table 2:** Interspecific genetic distances of COI sequences based on K2P model.

Species	Sample size (*n*)	1	2	3	4
(1) *Lignobrycon myersi*	59	—			
(2) *Triportheus guentheri*	4	0.174	—		
(3) *Astyanax fasciatus*	3	0.220	0.246	—	
(4) *Triportheus angulatus*	1	0.174	0.082	0.227	—
(5) *Triportheus nematurus*	4	0.183	0.094	0.240	0.049

*Mean*		*0.049*			

**Table 3:** Intraspecific genetic distance (*D*) and standard error (SE) of COI sequences based on K2P model.

Species	Sample size (*n*)	*D*	SE
*Lignobrycon myersi*	59	0.004	0.001
*Triportheus guentheri*	4	0.001	0.001
*Astyanax fasciatus*	3	0.005	0.002
*Triportheus angulatus*	1	n/c	n/c
*Triportheus nematurus*	4	0.000	0.000

*Mean*		*0.002*	

**Table 4:** Interpopulation genetic distance in COI sequences of *Lignobrycon myersi* based on K2P model.

Population	Sample size	1	2
(1) Almada River	14	—	
(2) Contas River	41	0.005	—
(3) Cachoeira River	4	0.013	0.013

**Table 5:** Haplotype (*h*) and nucleotide (*π*) diversity in COI sequences within populations of *Lignobrycon myersi*.

Population	Sample size	*h*	*π*
Almada River	14	0.396	0.002
Contas River	41	0.602	0.001
Cachoeira River	4	0.000	0.000

Total	59	0.774	0.004
